# Association between *ACTN3* (R577X), *ACE* (I/D), *BDKRB2* (-9/+9), and *AGT* (M268T) polymorphisms and performance phenotypes in Brazilian swimmers

**DOI:** 10.1186/s13102-024-00828-2

**Published:** 2024-02-19

**Authors:** Severino Leão de Albuquerque-Neto, Marcos Antonio Pereira dos Santos, Valmir Oliveira Silvino, Jose Juan Blanco Herrera, Thiago Santos Rosa, Glauber Castelo Branco Silva, Bruno Pena Couto, Cirley Pinheiro Ferreira, Alexandre Sérgio Silva, Sandro Soares de Almeida, Gislane Ferreira de Melo

**Affiliations:** 1 União Educacional do Nordeste (UNINORDESTE), João Pessoa, Paraíba, Brazil; 2 Department of Biophysics and Physiology, Nucleus of Study in Physiology Applied to Performance and Health (NEFADS), Federal University of Piaui, Teresina, Piauí Brazil; 3 Rede Nordeste de Biotecnologia (RENORBIO), Federal University of Piauí, Teresina, Piauí Brazil; 4 Brazilian Confederation of Aquatic Sports, Rio de Janeiro, Rio de Janeiro, Brazil; 5 Department of Physical Education, Catholic University of Brasília, Brasília, DF Brazil; 6 Department of Physical Education, State University of Piauí, Barros Araújo Campus, Picos, Piauí Brazil; 7 School of Health and Behavioural Sciences, The University of the Sunshine Coast, Sippy Downs, QLD Australia; 8 Associated Postgraduate Program in Physical Education of the Federal University of Pernambuco, Federal University of Paraíba, João Pessoa, Brazil; 9 Hospital Israelita Albert Einstein, São Paulo, São Paulo, Brazil; 10 Universidade Ibirapuera, São Paulo, São Paulo, Brazil; 11 Universidade Anhanguera, Guarulhos, São Paulo, Brazil

**Keywords:** Genetic polymorphisms, Strength, Aerobic performance, Swimming

## Abstract

**Background:**

The influence of genetic polymorphisms on athletic performance has been widely explored. This study investigated the interactions between the polymorphisms *ACTN3* (R577X), *ACE* (I/D), *BDKRB2* (-9/+9), and *AGT* (M/T) and their association with endurance and strength phenotypes in Brazilian swimmers.

**Methods:**

123 athletes (aged 20–30 years) and 718 controls participated in the study. The athletes were divided into elite and sub-elite (*N* = 19 and 104, respectively) and strength and endurance experts (*N* = 98 and 25, respectively). Hardy-Weinberg equilibrium was observed in all groups.

**Results:**

Considering the *ACE* polymorphism, it was observed a higher frequency of the DD genotype than expected in the strength experts of the elite group, whereas the strength experts sub-elite athletes had a higher frequency of the ID genotype (χ^2^ = 8.17; *p* = 0.01). Subjects with XX genotypes of *ACTN3* are more likely to belong to the athlete group when compared to the control group (OR = 1.79, *p* = 0.04). The DD homozygotes of the *ACE* are more likely to belong to the elite group with strength phenotypes than the group of sub-elite (OR = 7.96, *p* = 0.01) and elite strength experts compared to elite endurance (OR = 18.0, *p* = 0.03). However, no significant differences were observed in the allelic distribution of the polymorphisms evaluated when comparing Elite, sub-elite athletes and controls.

**Conclusion:**

*ACE* and *ACTN3* allele frequencies should be considered with regard to performance influencing factors in Brazilian swimmers.

**Supplementary Information:**

The online version contains supplementary material available at 10.1186/s13102-024-00828-2.

## Background

Sports performance is influenced by several factors, including interactions between genetic and environmental aspects that affect the variation and modulation of phenotypic characteristics [1]. Genotypic characteristics distinct elite athletes from non-athletes and even sub-elite athletes [2]. In the field of genetics applied to elite swimming, studies point out interactions between genotypic profiles of some candidate polymorphisms and strength/power phenotypes, associated with short-distance events (≤ 200 m) performed in Olympic-size swimming pools [3]. Likewise, genotypic profiles associated with resistance phenotypes are observed, commonly related to long events (≥ 400 m) performed in official pools or in open water [3, 4].

In the field of genetics applied to elite swimming, studies point out interactions between genotypic profiles of some candidate polymorphisms and strength/power phenotypes, associated with short-distance events (≤ 200 m) performed in Olympic-size swimming pools [3]. In short-distance swimming events, the alpha-actinin-3 (*ACTN3*) polymorphism is associated with power/strength phenotypes, with emphasis on the higher frequency of the R allele and the RR and RX genotypes [5]. The angiotensin-converting enzyme (*ACE*) I allele and II + ID genotypes are commonly associated with endurance phenotypes, especially among top-elite long-distance specialist athletes [3]. Regarding the bradykinin B2 receptor polymorphism (*BDKRB2*), -9 allele has been associated with endurance phenotypes [6], whereas + 9 was observed to be over-represented in short-distance in elite swimmers [7]. Similarly, it has been demonstrated a positive correlation between the angiotensinogen (*AGT*) M allele and the MT + MM genotypes and the resistance phenotypes, whereas the T allele has been associated with power/strength phenotypes [4].

To the best of our knowledge, there are no studies simultaneously investigating the association of *ACTN3* (R577X), *ACE* (I/D), *BDKRB2* (-9/+9), and *AGT* (M/T) polymorphisms with strength/power and endurance performance phenotypes in high-level Brazilian swimmers. Investigating the effect of the interaction between candidate polymorphisms and athletic performance in Brazilian swimmers can provide valuable insights into the genetic factors that may influence their abilities. Therefore, the main objective of the study was to identify and characterize the allelic and genotypic distribution of the aforementioned polymorphisms and their possible associations with Brazilian swimming athletes specialized in short (≤ 200 m) or in long events (≥ 400 m). It was hypothesized that the *ACTN3* (R577X), *ACE* (I/D), *BDKRB2* (-9/+9), and *AGT* (M/T) polymorphisms could influence the performance of swimming athletes.

## Methods

### Participants

A convenience sample was formed with 123 high-level Brazilian swimmers (76 men and 47 women aged between 20 and 30 years). They were initially divided by technical level and sporting experience (national and international) into an elite group (*n* = 19) and a sub-elite group (*n* = 104). The elite group was formed by athletes who represented the national team in international competitions, world championships and the Olympic Games, whereas the sub-elite group was formed by athletes who participated only in national-level championships. Later, the participants were also divided according to the swimming events at which they were expert. Short distance swimmers (≤ 200 m) were assigned to the power-strength phenotype (*n* = 98) and long-distance swimmers (≥ 400 m) were assigned to the endurance phenotype (*n* = 25). Only athletes between 18 and 40 years old affiliated with the Brazilian Confederation of Water Sports (BCWS) who signed the informed consent form were eligible for inclusion. Exclusion criteria were to be suffering from any chronic pathology or an injury in the month prior to the investigation, not to be affiliated with the BCWS during the study or not to give their informed written consent to participate. Finally, an age-matched control group (*n* = 718) composed of healthy non-athletes from Southwest and Central-west regions of Brazil representing the general population was formed.

Information regarding the main events attended by each athlete, as well as their technical index and experience (national and international), were verified on the BCWS official website (https://cbda.org.br/cbda/natacao/atletas). This study was carried out in accordance with the principles of the Declaration of Helsink and was approved in 2015 by the Research Ethics Committee of the Brasilia Catholic University, Brazil, under protocol 1.319.640. All subjects signed the informed consent form according to resolution 466/12 of the National Health Council.

### Genotyping

The genomic deoxyribonucleic acid (DNA) of the buccal mucosa cells were collected by scraping with a specific swab and the extraction was performed following the Chelex Resin protocol (BioRad Laboratories, Hercules, CA). DNA quantification and evaluation was performed in a NanoDrop® ND1000 spectrophotometer. The DNA used in the standard amplification reactions was diluted in autoclaved ultra-pure water.

The *ACTN3* (rs1815739) was genotyped by the allelic discrimination method in quantitative Polymerase Chain Reaction (qPCR) using TaqMan Assays by the high-performance QuantStudio™ 6 Flex Real-Time PCR System (Foster City, CA, USA). *ACE* polymorphism genotyping was performed as previously [8]. The *BDKRB2* genotype analysis was performed using 2 specific primers (forward 5’-TCCAGCTCTGGCTTCTGG − 3’ and reverse 5’- *AGT*CGCTCCCTGGTACTGC- 3’), in order to amplify and classify individuals as homozygotes (+ 9/+9 or -9/-9) or heterozygotes (+ 9/-9). *AGT* gene was examined using a polymerase chain reaction and the primer pairs used for its amplification were forward 5′CAGGGTGCTGTCCACACTGGACCCC′3′ and reverse 5′CCGTTTGTGCAGGGCCTGGCTCTCT′3.

After the genotype analysis step, total volume was loaded on a 2% agarose gel for the *ACE*, 3% for the *AGT*, and 4% for *BDKRB2*. Afterwards, the volume was subjected to electrophoresis for 30–35 min according to the expected amplicon patterns. The genotypes’ results were analyzed and interpreted using the iBright™ FL1500 Imaging System transilluminator (Thermo Fisher Scientific, USA). The visualization of the gene fragments after allelic discrimination and agarose gel electrophoresis can be seen in Supplementary File S1.

### Statistical analysis

The genotypic frequencies were initially compared among the athletes divided by sex through the Chi-square (χ ^2^) test. Since there was no difference between sex allowed all volunteers to be analyzed together. Subsequently, Hardy-Weinberg equilibrium was verified between athletes in each of the four polymorphisms [9]. The genotypic frequencies of the athletes and controls were compared via the Chi-square test. 2 × 2 tables were used to compare the allelic frequency between groups for each polymorphism. In all comparisons, opposing phenotypic groups (power/strength vs. endurance) and sport status (elite vs. sub-elite) were considered. For all chi-square analyses, effect sizes were measured with the Cramer’s V statistic [10]. The Chi-square test was conditioned to the interpretation of the residue (R = observed value minus the expected value) and the adjusted residuals. The residual analysis allows pointing out which category of the group presents a significant value (positive value) and to determine the level of significance for the excess of occurrences through the adjusted residual. Thus, an adjusted residual greater than 1.96 indicated that the value was significantly larger than would be expected if the null hypothesis were true, with a significance level of 0.05. Additionally, the correspondence analysis test was performed to analyze the relationship between the categorical variables. Binary logistic regression was used to assess the odds ratio (OR) of the athletes with a certain genotypic characteristic belonging to the elite or sub-elite group considering codominant (1/1 vs. 1/2 and 1/1 vs. 2/2), dominant (1/1 vs. 1/2 + 2/2) and recessive (1/1 + 1/2 vs. 2/2) models. The significance level adopted for the statistical procedures was *p* ≤ 0.05. The statistical analysis was performed using the SPSS 22.0 software for Windows (IBM, USA).

## Results

The genotypic distribution and allelic frequency for the *ACTN3* (R577X), *ACE* (I/D), *BDKRB2* (-9/+9) and *AGT* (M268T) polymorphisms are presented in Table 1. All genotypes were in Hardy-Weinberg equilibrium (*p* > 0.05).

**Table 1:** Genotypic and allelic frequencies of all the groups evaluated

Genotype	Controls	All athletes	Elite athletes	Elite power/strength	Elite endurance	Sub-elite athletes	Sub-elite power/strength	Sub-elite endurance
***ACTN3***								
RR	162 (36.0%)	35 (28.5%)	3 (15.8%)	2 (15.4%)	1 (16.7%)	32 (30.8%)	25 (29.4%)	7 (36.8%)
RX	205 (45.6%)	56 (45.5%)	10 (52.6%)	8 (61.5%)	2 (33.3%)	46 (44.2%)	38 (44.7%)	8 (42.1%)
XX	83 (18.4%)	32 (26.0%)	6 (31.6%)	3 (23.1%)	3 (50.0%)	26 (25.0%)	22 (25.9%)	4 (21.1%)
Total	450	123	19	13	6	104	85	19
R allele (wild)	367 (56.0%)	91 (50.8%)	13 (44.8%)	10 (47.6%)	3 (37.5%)	78 (52.0%)	63 (51.2%)	15 (55.6%)
X allele (mutant)	288 (44.0%)	88 (49.2%)	16 (55.2%)	11 (52.4%)	5 (62.5%)	72 (48.0%)	60 (48.8%)	12 (44.4%)
***ACE***								
II	138 (19.2%)	19 (15.4%)	3 (15.8%)	2 (15.4%)	1 (16.7%)	16 (15.4%)	13 (15.3%)	3 (15%)
ID	341 (47.5%)	62 (50.4%)	6 (31.6%)	2 (15.4%)	4 (66.7%)	56 (53.8%)	46 (54.1%)	11 (55%)
DD	239 (33.3%)	42 (34.1%)	10 (52.6%)	9 (69.2%)	1 (16.7%)	32 (30.8%)	26 (30.6%)	6 (30%)
Total	718	123	19	13	6	104	85	20
D allele (wild)	580 (54.8%)	104 (56.2%)	16 (64.0%)	11 (73.3%)	5 (50.0%)	88 (55.0%)	72 (58.1%)	16 (55.2%)
I allele (mutant)	479 (45.2%)	81 (43.8%)	9 (36.0%)	4 (26.7%)	5 (50.0%)	72 (45.0%)	52 (41.9%)	13 (44.8%)
***BDKRB2***								
+ 9/+9	154 (27.1%)	34 (27.6%)	5 (26.3%)	2 (15.4%)	3 (50.0%)	29 (27.9%)	23 (27.1%)	6 (30.0%)
-9/+9	284 (50.1%)	62 (50.4%)	8 (42.1%)	6 (46.2%)	2 (33.3%)	54 (51.9%)	43 (50.6%)	11 (60.0%)
-9/-9	129 (22.8%)	27 (22.0%)	6 (31.6%)	5 (38.5%)	1 (16.7%)	21 (20.2%)	19 (22.4%)	2 (10.0%)
Total	567	123	19	13	6	104	85	20
+ 9 allele (wild)	483 (53.9%)	96 (51.6%)	13 (48.1%)	8 (42.1%)	5 (62.5%)	83 (52.5%)	66 (51.6%)	17 (56.7%)
-9 allele (mutant)	413 (46.1%)	89 (48.1%)	14 (51.9%)	11 (57.9%)	3 (37.5%)	75 (47.5%)	62 (48.4%)	13 (43.3%)
***AGT***								
MM	72 (31.2%)	41 (36.0%)	9 (52.9%)	6 (54.5%)	3 (50.0%)	32 (33.0%)	27 (34.2%)	6 (31.6%)
MT	112 (48.5%)	47 (41.2%)	6 (35.3%)	3 (27.3%)	3 (50.0%)	41 (42.3%)	32 (40.5%)	9 (47.4%)
TT	47 (20.3%)	26 (22.8%)	2 (11.8%)	2 (18.2%)	0 (0.0%)	24 (24.7%)	20 (25.3%)	4 (21.1%)
Total	231	114	17	11	6	97	79	19
M allele (wild)	184 (53.6%)	88 (54.7%)	15 (65.2%)	9 (64.3%)	6 (66.7%)	73 (52.9%)	59 (53.2%)	14 (51.9%)
T allele (mutant)	159 (46.4%)	73 (45.3%)	8 (34.8%)	5 (35.7%)	3 (33.3%)	65 (47.1%)	52 (46.8%)	13 (48.1%)

Note: Genotype distribution among all tested controls and athletes was in Hardy-Weinberg equilibrium for the *ACTN3* (controls, *p* = 0.20; athletes, *p* = 0.32), *ACE* (controls, *p* = 0.41; athletes, *p* = 0.62),*BDKRB2* (controls, *p* = 0.93; athletes, *p* = 0.90), and *AGT* (controls, *p* = 0.77; athletes, *p* = 0.09)

The genotypic distribution of swimmers for the polymorphisms analyzed was not significantly different between the elite, sub-elite, and control groups (Table 2). However, when comparing sub-elite and elite athletes with power/strength phenotypes, we found a statistically significant difference for *ACE* polymorphism (*p* = 0.017).

**Table 2:** Comparisons between the genotype frequencies of the studied groups via 2 × 2 Chi-square test

Polymorphisms (Alleles 1/2)	Groups	n	Genotypes			Genotypic comparison	χ ^2^ (2 × 2) (df = 2)	*p*	Cramer’s V
			1/1	1/2	2/2				
***ACTN3*** (R/X)	Controls	450	162 (36.0%)	205 (45.6%)	83 (18.4%)	Control vs. Elite	3.97	0.137	0.092
	All athletes	123	35 (28.5%)	56 (45.5%)	32 (26.0%)	Control vs. All athletes	4.36	0.113	0.087
	Sub-elite	104	32 (30.8%)	46 (44.2%)	26 (25.0%)	Control vs. Sub-elite	2.54	0.218	0.068
	Elite	19	3 (15.8%)	10 (52.6%)	6 (31.6%)	Elite vs. Sub-elite	1.78	0.410	0.120
	Sub-elite Pow	85	25 (29.4%)	38 (44.7%)	22 (25.9%)	Sub-elite Pow vs. Elite Pow	1.52	0.468	0.120
	Sub-elite End	19	7 (36.8%)	8 (42.1%)	4 (21.1%)	Sub-elite End vs. Sub-elite Pow	1.03	0.596	0.100
	Elite Pow	13	2 (15.4%)	8 (61.5%)	3 (23.1%)	Elite Pow vs. Elite End	1.57	0.457	0.290
	Elite End	6	1 (16.7%)	2 (33.3%)	3 (50.0%)	Elite End vs. Sub-elite End	2.03	0.362	0.280
***ACE*** (I/D)	Controls	718	138 (19.2%)	341 (47.5%)	239 (33.3%)	Control vs. Elite	3.16	0.206	0.065
	All athletes	123	19 (15.4%)	62 (50.4%)	42 (34.1%)	Control vs. All athletes	1.01	0.604	0.035
	Sub-elite	104	16 (15.4%)	56 (53.8%)	32 (30.8%)	Control vs. Sub-elite	1.65	0.439	0.045
	Elite	19	3 (15.8%)	6 (31.6%)	10 (52.6%)	Elite vs. Sub-elite	3.83	0.147	0.180
	Sub-elite Pow	85	13 (15.3%)	46 (54.1%)	26 (30.6%)	Sub-elite Pow vs. Elite Pow	8.17	0.017*	0.290
	Sub-elite End	20	3 (15%)	11 (55%)	6 (30%)	Sub-elite End vs. Sub-elite Pow	0.01	0.993	0.010
	Elite Pow	13	2 (15.4%)	2 (15.4%)	9 (69.2%)	Elite Pow vs. Elite End	5.58	0.061	0.540
	Elite End	6	1 (16.7%)	4 (66.7%)	1 (16.7%)	Elite End vs. Sub-elite End	0.53	0.769	0.140
***BDKRB2*** (-9/+9)	Controls	567	129 (22.8%)	346 (50.1%)	154 (27.1%)	Control vs. Elite	0.47	0.791	0.028
	All athletes	123	27 (22.0%)	62 (50.4%)	34 (27.6%)	Control vs. All athletes	2.07	0.355	0.055
	Sub-elite	104	21 (20.2%)	54 (51.9%)	29 (27.9%)	Control vs. Sub-elite	2.68	0.262	0.063
	Elite	19	6 (31.6%)	8 (42.1%)	5 (26.3%)	Elite vs. Sub-elite	1.27	0.530	0.100
	Sub-elite Pow	85	19 (22.4%)	43 (50.6%)	23 (27.1%)	Sub-elite Pow vs. Elite Pow	1.84	0.398	0.140
	Sub-elite End	20	2 (10.0%)	11 (60.0%)	6 (30.0%)	Sub-elite End vs. Sub-elite Pow	1.62	0.444	0.120
	Elite Pow	13	5 (38.5%)	6 (46.2%)	2 (15.4%)	Elite Pow vs. Elite End	2.65	0.266	0.370
	Elite End	6	1 (16.7%)	2 (33.3%)	3 (50.0%)	Elite End vs. Sub-elite End	1.10	0.576	0.210
***AGT*** (M/T)	Controls	231	72 (31.2%)	112 (48.5%)	47 (20.3%)	Control vs. Elite	3.47	0.177	0.120
	All athletes	114	41 (36.0%)	47 (41.2%)	26 (22.8%)	Control vs. All athletes	1.63	0.443	0.069
	Sub-elite	97	32 (33.0%)	41 (42.3%)	24 (24.7%)	Control vs. Sub-elite	1.25	0.536	0.062
	Elite	17	9 (52.9%)	6 (35.3%)	2 (11.8%)	Elite vs. Sub-Elite	2.84	0.242	0.160
	Sub-elite Pow	79	27 (34.2%)	32 (40.5%)	20 (25.3%)	Sub-elite Pow vs. Elite Pow	1.73	0.421	0.140
	Sub-elite End	19	6 (31.6%)	9 (47.4%)	4 (21.1%)	Sub-elite End vs. Sub-elite Pow	0.29	0.865	0.054
	Elite Pow	11	6 (54.5%)	3 (27.3%)	2 (18.2%)	Elite Pow vs. Elite End	1.67	0.433	0.310
	Elite End	6	3 (50.0%)	3 (50.0%)	0 (0.0%)	Elite End vs. Sub-elite End	2.00	0.368	0.260

df, degrees of freedom; End, endurance; Pow, power/strength

The adjusted residue analysis verified that the observed frequency of the DD genotype was higher than expected in the power/strength elite group. In contrast, the sub-elite athletes with power/strength phenotypes presented a higher frequency of the ID genotype than expected (χ^2^ = 8.168; *p* = 0.013) (Table 3).

**Table 3:** Analysis of *ACE* polymorphism in the Elite power/strength vs. Sub-elite power/strength comparison

	Genotypes	Elite power/strength	AR	Sub-elite power/strength	AR	χ ^2^	*p*	Cramer’s V
*ACE*	DD	9 (69.2%)	2.7	26 (30.6%)	-2.7	8.168	0.013	0.289
	ID	2 (15.4%)	-2.6	46 (54.1%)	2.6			
	II	2 (15.4%)	0	13 (15.3%)	0			

Data presented as percentage (%). AR, adjusted and standardized residue; χ^2^, Chi–square

**Fig. 1:**
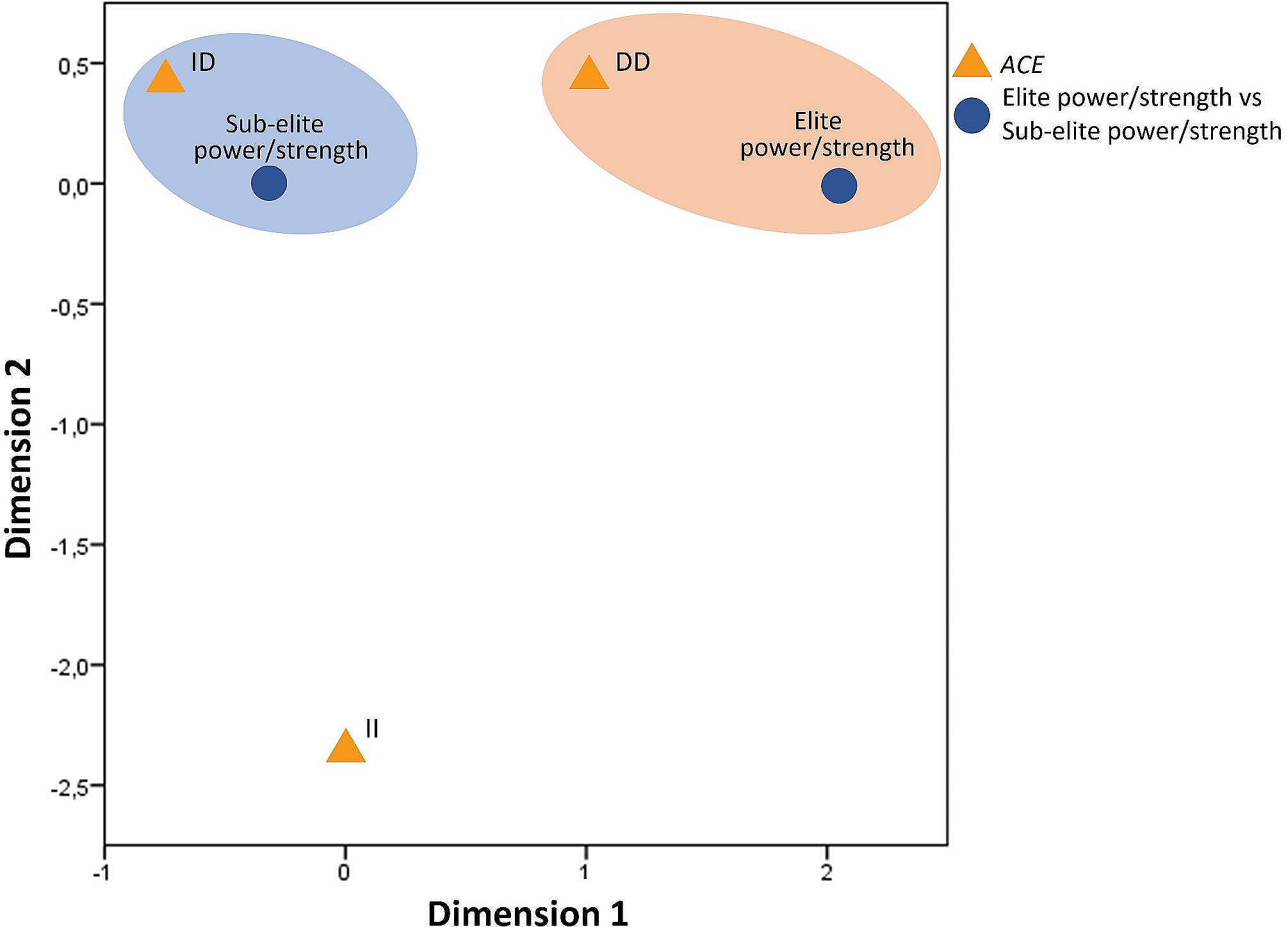
Correspondence analysis of the *ACE* genotypes in relation to the elite power/strength and sub-elite power/strength athletes

Figure 1 presents the correspondence analysis between the *ACE* genotypes in relation to the elite power/strength athletes vs. sub-elite power/strength athletes. This figure was generated from the adjusted residual data presented in Table 3, which shows a greater association between the DD genotype and the elite power/strength group and the ID genotype with the sub-elite power/strength group.

Figure 1. Correspondence analysis of the *ACE* genotypes in relation to the elite power/strength and sub-elite power/strength athletes.

The allelic frequencies and possible comparisons between groups are shown in Table 4. No significant differences were observed in the allelic distribution of the studied polymorphisms when comparing elite athletes, sub-elite athletes, and controls. Finally, Table 5 shows the comparisons with Odds Ratio between groups regarding genotypic distribution via codominant, dominant, and recessive models.

**Table 4:** Comparisons between the allelic frequencies of the studied groups

Polymorphisms (Alleles 1/2)	Groups	Alleles (%)		Allelic comparisons	χ^2^ (2 × 2) (df = 1)	*p*	Cramer’s V
		1	2				
***ACTN3*** (R/X)	Controls	367 (56.0%)	288 (44.0%)	Elite vs. Control	1.412	0.235	0.045
	All athletes	91 (50.8%)	88 (49.2%)	All athletes vs. Control	1.531	0.216	0.043
	Sub-elite	78 (52.0%)	72 (48%)	Sub-elite vs. Control	0.802	0.370	0.032
	Elite	13 (44.8%)	16 (55.2%)	Elite vs. Sub-elite	0.500	0.479	0.053
	Sub-elite Pow	63 (51.2%)	60 (48.8%)	Sub-elite Pow vs. Elite Pow	0.093	0.760	0.025
	Sub-elite End	15 (55.6%)	12 (44.4%)	Sub-elite End vs. Sub-elite Pow	0.167	0.683	0.033
	Elite Pow	10 (47.6%)	11 (52.4%)	Elite Pow vs. Elite End	0.240	0.624	0.091
	Elite End	3 (37.5%)	5 (62.5%)	Elite End vs. Sub-elite End	0.805	0.369	0.150
***ACE*** (I/D)	Controls	479 (45.2%)	580 (54.8%)	Elite vs. Control	0.841	0.359	0.028
	All athletes	81 (43.8%)	104 (56.2%)	All athletes vs. Control	0.133	0.715	0.010
	Sub-elite	72 (45.0%)	88 (55.0%)	Sub-Elite vs. Control	0.003	0.956	0.002
	Elite	9 (36.0%)	16 (64.0%)	Elite vs. Sub-elite	0.712	0.399	0.062
	Sub-elite Pow	52 (41.9%)	72 (58.1%)	Sub-elite Pow vs. Elite Pow	1.297	0.255	0.097
	Sub-elite End	13 (44.8%)	16 (55.2%)	Sub-elite End vs. Sub-elite Pow	0.080	0.777	0.023
	Elite Pow	4 (26.7%)	11 (73.3%)	Elite Pow vs. Elite End	1.418	0.234	0.240
	Elite End	5 (50.0%)	5 (50.0%)	Elite End vs. Sub-elite End	0.080	0.777	0.045
***BDKRB2*** (-9/+9)	Controls	413 (46.1%)	483 (53.9%)	Elite vs. Control	0.236	0.627	0.016
	All athletes	89 (48.1%)	96 (51.6%)	All athletes vs. Control	0.250	0.617	0.015
	Sub-elite	75 (47.5%)	83 (52.5%)	Sub-Elite vs. Control	0.102	0.749	0.010
	Elite	14 (51.9%)	13 (48.1%)	Elite vs. Sub-elite	0.177	0.674	0.031
	Sub-elite Pow	62 (48.4%)	66 (51.6%)	Sub-elite Pow vs. Elite Pow	0.592	0.442	0.063
	Sub-elite End	13 (43.3%)	17 (56.7%)	Sub-elite End vs. Sub-elite Pow	0.254	0.614	0.040
	Elite Pow	11 (57.9%)	8 (42.1%)	Elite Pow vs. Elite End	0.938	0.333	0.190
	Elite End	3 (37.5%)	5 (62.5%)	Elite End vs. Sub-elite End	0.088	0.767	0.048
***AGT*** (M/T)	Controls	184 (53.6%)	159 (46.4%)	Elite vs. Control	1.164	0.281	0.056
	All athletes	88 (54.7%)	73 (45.3%)	All athletes vs. Control	0.045	0.831	0.010
	Sub-elite	73 (52.9%)	65 (47.1%)	Sub-Elite vs. Control	0.022	0.882	0.007
	Elite	15 (65.2%)	8 (34.8%)	Elite vs. Sub-elite	1.207	0.272	0.087
	Sub-elite Pow	59 (53.2%)	52 (46.8%)	Sub-elite Pow vs. Elite Pow	0.621	0.431	0.070
	Sub-elite End	14 (51.9%)	13 (48.1%)	Sub-elite End vs. Sub-elite Pow	0.015	0.903	0.010
	Elite Pow	9 (64.3%)	5 (35.7%)	Elite Pow vs. Elite End	0.014	0.907	0.024
	Elite End	6 (66.7%)	3 (33.3%)	Elite End vs. Sub-elite End	0.600	0.439	0.130

df, degrees of freedom; End, Endurance; Pow, power/strength; χ^2^, Chi–square

**Table 5:** Comparisons via binary logistic regression with Odds Ratio between groups regarding genotypic distribution through codominant (1/1 vs. 1/2 and 1/1 vs. 2/2), dominant (1/1 vs. 1/2 + 2/2) and recessive (1/1 + 1/2 vs. 2/2) models

Polymorphisms (Allele 1/2)	Comparisons	1/1 vs. 1/2	1/1 vs. 2/2	1/1 vs. 1/2 + 2/2	1/1 + 1/2 vs. 2/2
		OR (95% CI); p	OR (95% CI); p	OR (95% CI); p	OR (95% CI); p
*ACTN3* (R/X)	Elite vs. Control	n.s.	n.s.	n.s.	n.s.
	All athletes vs. Control	n.s.	**1.79 (1.03–3.09); 0.04**	n.s.	n.s.
	Sub-elite vs. Control	n.s.	n.s.	n.s.	n.s.
	Elite vs. Sub-elite	n.s.	n.s.	n.s.	n.s.
	Sub-elite Pow vs. Elite Pow	n.s.	n.s.	n.s.	n.s.
	Sub-elite End vs. Sub-elite Pow	n.s.	n.s.	n.s.	n.s.
	Elite Pow vs. Elite End	n.s.	n.s.	n.s.	n.s.
	Elite End vs. Sub-elite End	n.s.	n.s.	n.s.	n.s.
*ACE* (I/D)	Elite vs. Control	n.s.	n.s.	n.s.	n.s.
	All athletes vs. Control	n.s.	n.s.	n.s.	n.s.
	Sub-elite vs. Control	n.s.	n.s.	n.s.	n.s.
	Elite vs. Sub-elite	n.s.	n.s.	n.s.	n.s.
	Sub-elite Pow vs. Elite Pow	**7.96 (1.60–39.66); 0.01**	n.s.	**5.10 (1.44–18.9); 0.01**	n.s.
	Sub-elite End vs. Sub-elite Pow	n.s.	n.s.	n.s.	n.s.
	Elite Pow vs. Elite End	**18.0 (1.24–260.88); 0.03**	n.s.	n.s.	n.s.
	Elite End vs. Sub-elite End	n.s.	n.s.	n.s.	n.s.
*BDKRB2* (-9/+9)	Elite vs. Control	n.s.	n.s.	n.s.	n.s.
	All athletes vs. Control	n.s.	n.s.	n.s.	n.s.
	Sub-elite vs. Control	n.s.	n.s.	n.s.	n.s.
	Elite vs. Sub-elite	n.s.	n.s.	n.s.	n.s.
	Sub-elite Pow vs. Elite Pow	n.s.	n.s.	n.s.	n.s.
	Sub-elite End vs. Sub-elite Pow	n.s.	n.s.	n.s.	n.s.
	Elite Pow vs. Elite End	n.s.	n.s.	n.s.	n.s.
	Elite End vs. Sub-elite End	n.s.	n.s.	n.s.	n.s.
*AGT* (M/T)	Elite vs. Control	n.s.	n.s.	n.s.	n.s.
	All athletes vs. Control	n.s.	n.s.	n.s.	n.s.
	Sub-elite vs. Control	n.s.	n.s.	n.s.	n.s.
	Elite vs. Sub-elite	n.s.	n.s.	n.s.	n.s.
	Sub-elite Pow vs. Elite Pow	n.s.	n.s.	n.s.	n.s.
	Sub-elite End vs. Sub-elite Pow	n.s.	n.s.	n.s.	n.s.
	Elite Pow vs. Elite End	n.s.	n.s.	n.s.	n.s.
	Elite End vs. Sub-elite End	n.s.	n.s.	n.s.	n.s.

CI, confidence interval; End, Endurance; n.s., not significant; OR, odds ratio; Pow, power/strength

## Discussion

Competitive performance of swimmers is the result of the complex interaction between environmental, nutritional, physical, physiological, biomechanical, sociocultural and genetic factors [11, 12]. Regarding the genetic components, it is a challenging task to recognize specific markers that affect physical performance in elite sport [13]. In swimming, endurance and power/strength phenotypes are essential for athletes to stand out at an excellency level [3, 14].

The majority of studies involving candidate polymorphisms related to swimming performance have given more attention to either *ACTN3* or *ACE* polymorphisms [3, 5, 14–19]. In addition, other polymorphisms have also been investigated, such as the *NOS3* genes (G894T and − 786T/C) [20], insulin-like growth factor (*IGF*) [21], myostatin (*MSTN*) [22], as well as interleukin-6 (IL6), MCT1, peroxisome proliferator-activated receptor alpha (*PPARA*), *PPARG* coactivator 1 alpha (*PPARGC1A*) and vascular endothelial growth factor receptor 2 (*VEGFR2*) [23, 24].

This study raised the hypothesis that the *ACTN3* (R577X), *ACE* (I/D), *BDKRB2* (-9/+9), and *AGT* (M/T) polymorphisms could influence the physical performance of Brazilian swimmers. Previous investigations demonstrated that the *ACE* (I/D) polymorphisms may be associated with both sprint/power and endurance performances [25]. Our study observed an association between the *ACE* gene polymorphism and the performance of swimmers when comparing elite and sub-elite Brazilian swimmers with power/strength phenotypes, experts in short distance events (≤ 200 m). Furthermore, in the elite power/strength group, the frequency of the DD genotype was greater than expected. In contrast, the frequency of the ID genotype observed was higher than expected in the sub-elite power/strength group.

The results obtained in the present study are in line with the findings by Costa et al. [26], who found a higher frequency of the DD genotype in elite short-distance swimmers when compared to the control group and endurance swimmers (≥ 400 m). In fact, it has been suggested that the DD genotype can provide possible advantages in athletic performance when analyzing athletes from various sports at national and/or international levels, including swimming [27]. Furthermore, the *ACE* DD genotype has been demonstrated to be beneficial in short duration aerobic exercises (2–8 min) when associated with training [28]. Likewise, the presence of DD homozygotes is suggested to be advantageous for power/strength performance in sprinters and jumpers compared to endurance athletes [29].

Several studies support the association between *ACE* and sports performance [30, 31]. In our results, it was verified that athletes of the power/strength group with the *ACE* DD genotype are 7.96 times more likely to belong to the groups of elite athletes when compared to sub-elite athletes. However, the odds increase 18 times among elite swimmers with DD genotype of the power/strength group when compared to elite swimmers with endurance phenotypes. We also observed that *ACE* DD homozygotes are 5.1 times more likely to belong to the elite athletes of the power/strength group than the ID + II genotypes compared to sub-elite athletes with the same phenotypic characteristic.

Previous studies suggest that individuals with a higher frequency of the *ACE* DD genotype have greater strength/power than those with genotype II and ID overexpression [32]. A recent meta-analysis study found that the predominance of *ACE* genotype II is indicated as advantageous for the performance of individuals aiming for endurance performance [33]. In contrast, ID genotype is more frequently expressed among athletes who practice sports that require high aerobic activity [34].

The *ACTN3* polymorphism has been extensively studied and associated with elite athletic performance [35, 36], including among swimming athletes [3, 5, 16, 18, 37]. The results of the present study showed that, regarding the *ACTN3* gene, individuals with the XX genotype are 1.79 times more likely to belong to the athlete group when compared to the control group. According to Ahmetov et al. [38], the homozygous XX genotype of *ACTN3* were significantly over-represented among endurance runners when compared to the control group, suggesting the need for both power and speed components for success in various endurance-oriented sporting events. Other studies have also shown that the XX genotype of *ACTN3* has been associated with athlete status, especially in sports with endurance nature [18].

Although the different genotypes of *ACE* and *ACTN3* polymorphisms influence the performance of athletes from different sport disciplines, these associations are not strong enough to predict the phenotypic characteristics of elite swimming athletes. Furthermore, the results of this study need to be interpreted considering the context of ethnic differences [39]. This study is limited by the small number of polymorphisms within a small and admixtured cohort. The data regarding individual genes lack a comprehensive analysis, and our study does not explore the more in-depth insights that could be obtained by considering haplotypes of several genes [40]. Therefore, further studies with different populations and a greater sample size would be suggested, since, to the best of our knowledge, this is the first study to verify the allele and genotypic frequency of the polymorphisms *ACTN3* (R577X), *ACE* (I/D), *BDKRB2* (-9/+9) and *AGT* (M268T) with Brazilian elite and sub-elite swimmers.

## Conclusion

The findings of the present study provide relevant considerations regarding the identification of strength/power and endurance talents in swimming. Elite and sub-elite athletes showed similar genotype frequencies to non-athletes. Our results indicate that the allele and genotype frequency of *ACE* and *ACTN3* should be taken into account as possible candidates for sports performance in swimming athletes. On the other hand, we could not observe the same interaction regarding the polymorphisms of the *AGT* and *BDKRB2* genes for the same sample group.

### Electronic supplementary material

Below is the link to the electronic supplementary material.

Supplementary Material 1

## Data Availability

The datasets generated and/or analyzed during the current study are available in the Figshare repository and can be found at 10.6084/m9.figshare.24913146.

## Abbreviations

ACE: Angiotensin-converting enzyme
ACTN3: Alpha-actinin-3
AGT: Angiotensinogen
BDKRB2: Bradykinin B2 receptor
DNA: Deoxyribonucleic acid
IGF: Insulin-like growth factor
IL6: Interleukin-6
MSTN: Myostatin
OR: Odds ratio
PPARA: Peroxisome proliferator-activated receptor alpha
PPARGC1A: PPARG coactivator 1 alpha
VEGFR2: Vascular endothelial growth factor receptor 2
χ2: Chi-square
