# Supplementary material for: Association between ACTN3 (R577X), ACE (I/D), BDKRB2 (-9/+9), and AGT (M268T) polymorphisms and performance phenotypes in Brazilian swimmers
Source: BMC Sports Sci Med Rehabil. 2024 Feb 19;16:50. doi: 10.1186/s13102-024-00828-2 (PMC10877830; doi:10.1186/s13102-024-00828-2)

## Slide 1
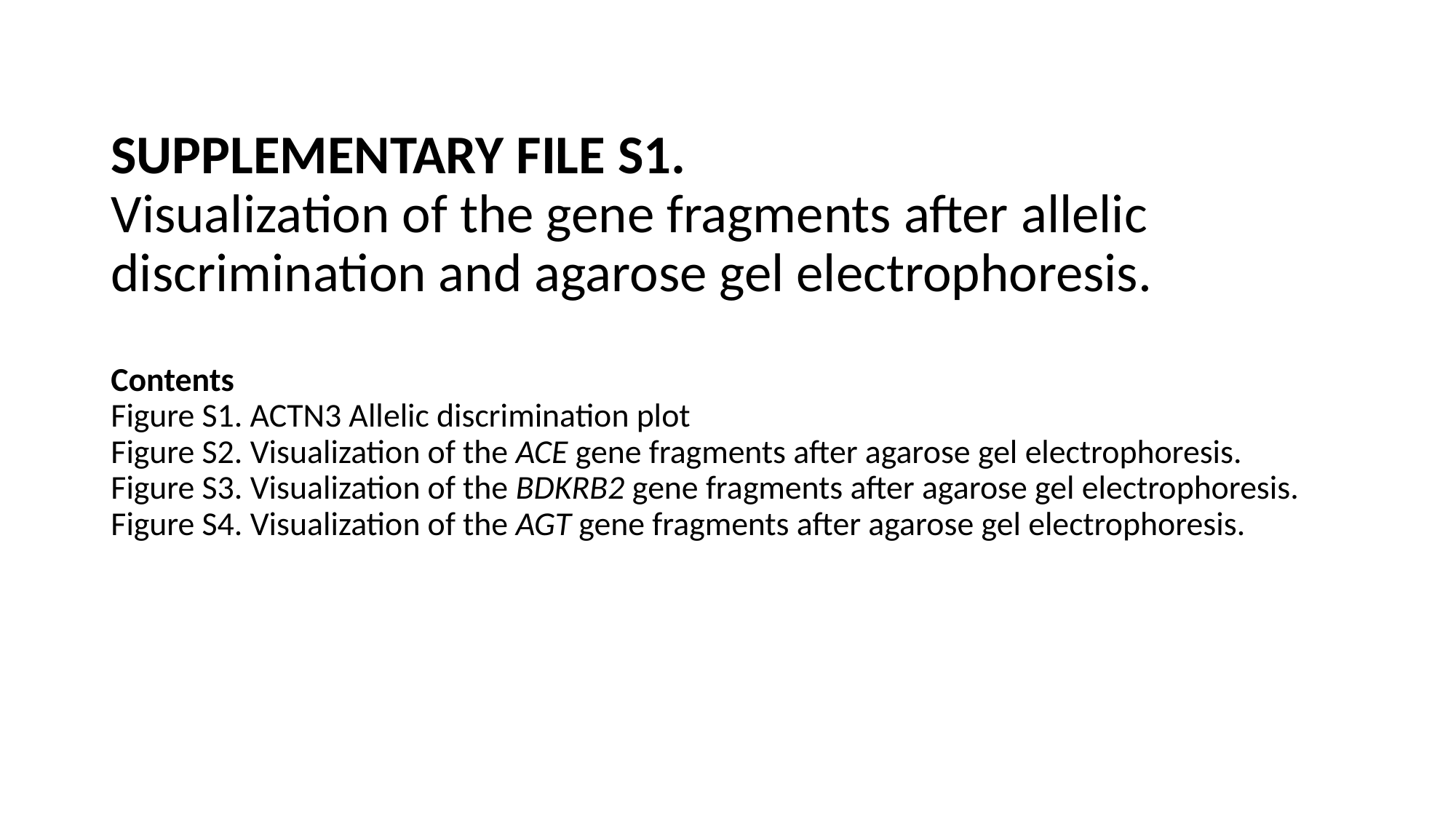

# SUPPLEMENTARY FILE S1. Visualization of the gene fragments after allelic discrimination and agarose gel electrophoresis. Contents Figure S1. ACTN3 Allelic discrimination plot Figure S2. Visualization of the ACE gene fragments after agarose gel electrophoresis. Figure S3. Visualization of the BDKRB2 gene fragments after agarose gel electrophoresis. Figure S4. Visualization of the AGT gene fragments after agarose gel electrophoresis.

## Slide 2
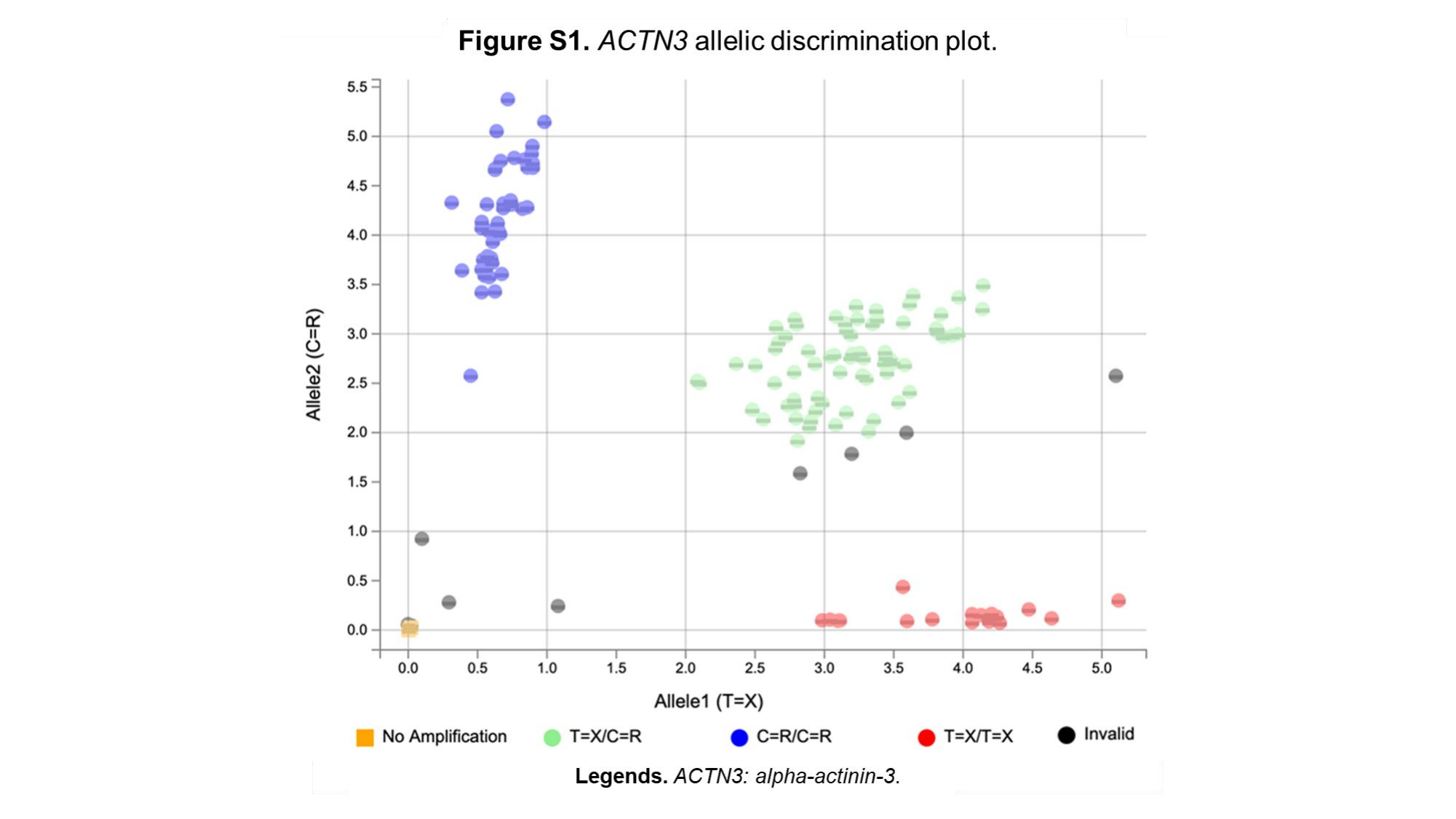

## Slide 3
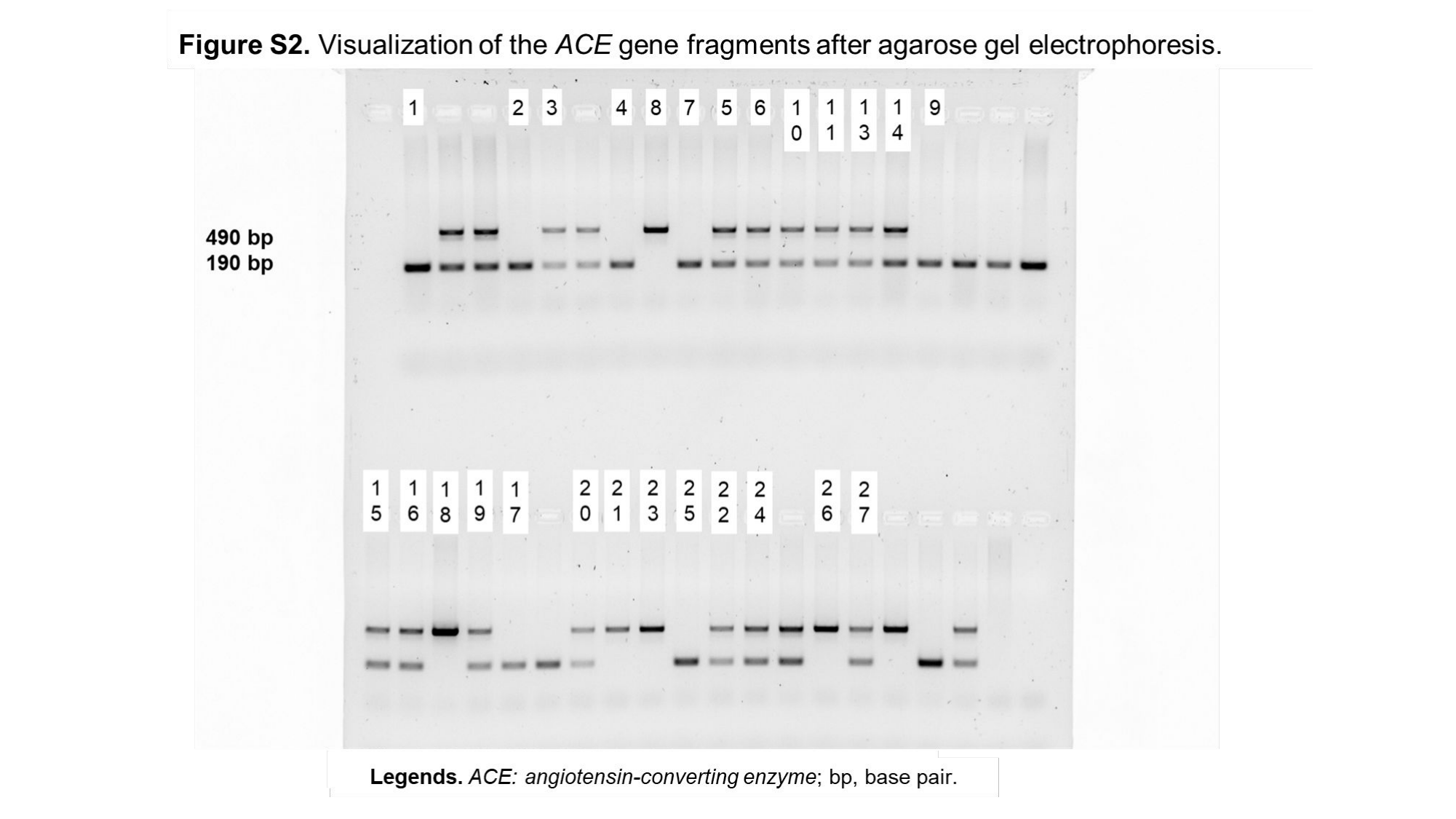

## Slide 4
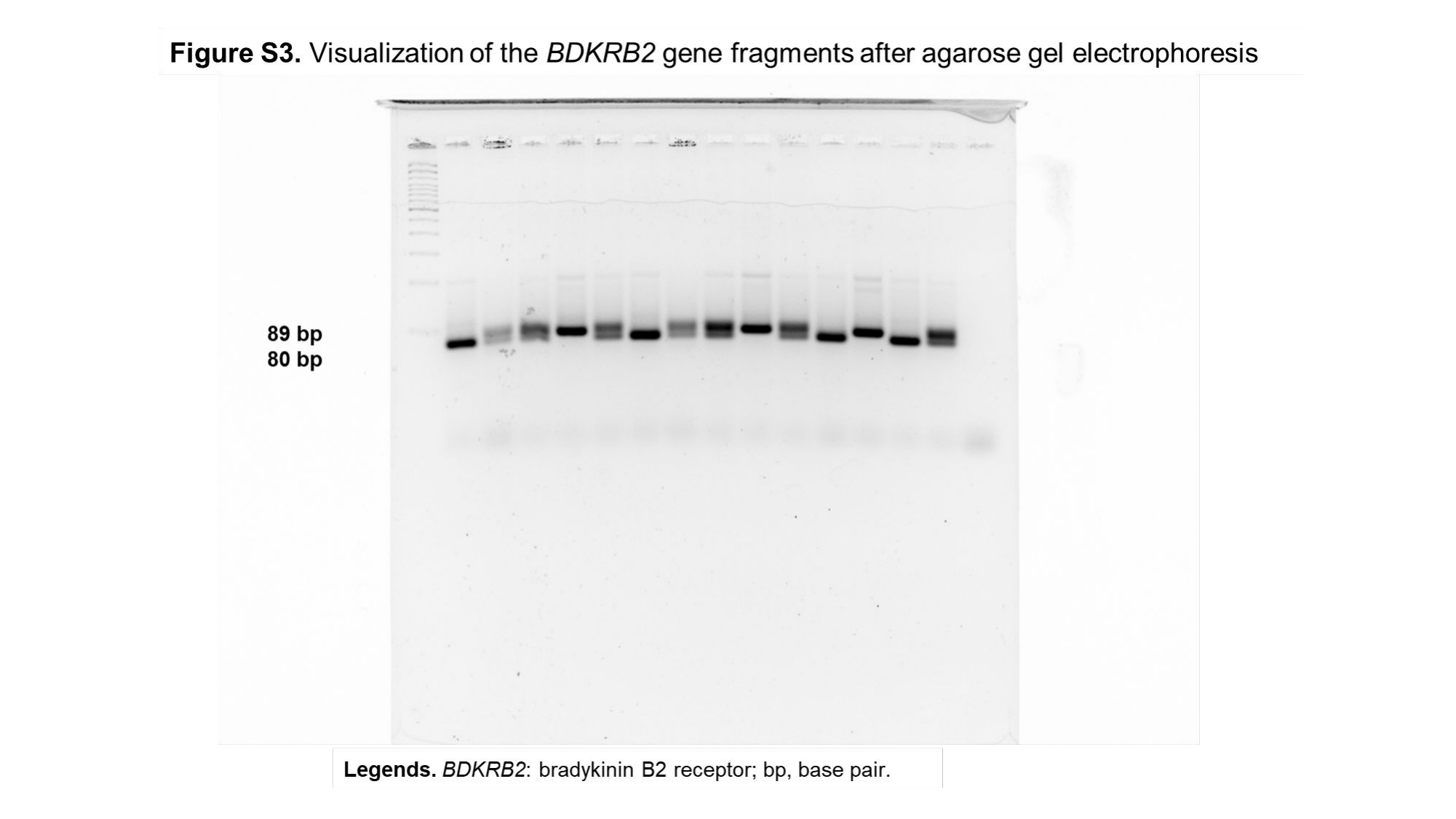

## Slide 5
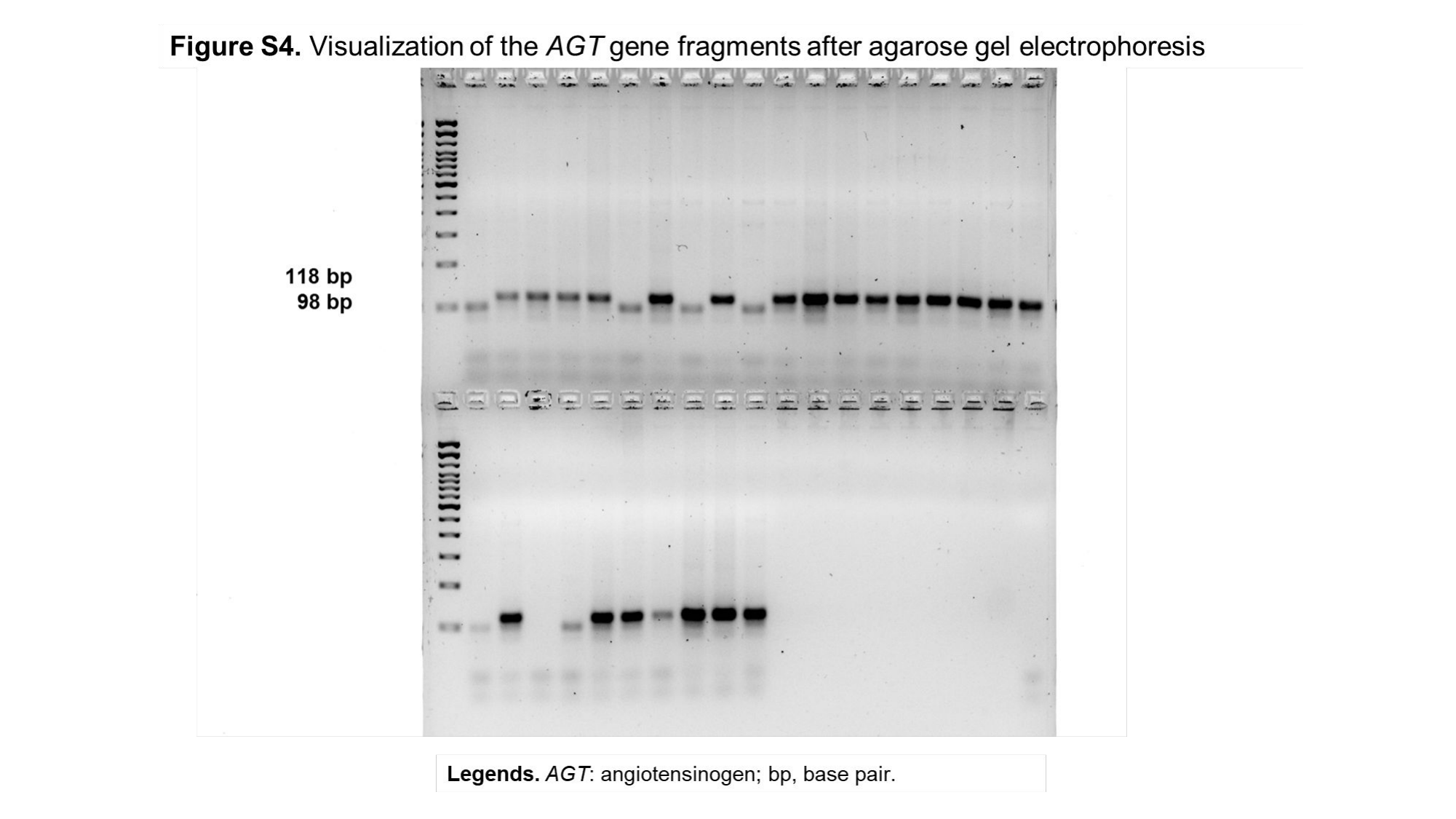

Supplement: Supplementary file 1 — Supplementary Material 1 [file 13102_2024_828_MOESM1_ESM.pptx]
